# Implications of partial culling on African swine fever control effectiveness in Vietnam

**DOI:** 10.3389/fvets.2022.957918

**Published:** 2022-09-06

**Authors:** Bui Thi To Nga, Pawin Padungtod, Klaus Depner, Vo Dinh Chuong, Do Tien Duy, Nguyen Duc Anh, Klaas Dietze

**Affiliations:** ^1^Department of Veterinary Medicine, Faculty of Veterinary Medicine, Vietnam National University of Agriculture, Hanoi, Vietnam; ^2^Food and Agriculture Organization of the United Nations (FAO), Country Office for Vietnam, Hanoi, Vietnam; ^3^Institute of International Animal Health/One Health, Friedrich-Loeffler-Institut, Greifswald, Germany; ^4^Department of Animal Health, Ministry of Agriculture and Rural Development, Hanoi, Vietnam; ^5^Department of Infectious Diseases and Veterinary Public Health, Faculty of Animal Science and Veterinary Medicine, Nong Lam University, Ho Chi Minh City, Vietnam

**Keywords:** disease management, culling policies, outbreak epidemiology, smallholder, pig production, acceptance

## Abstract

The introduction of the African swine fever (ASF) into previously unaffected countries often overwhelms veterinary authorities with the resource demanding control efforts that need to be undertaken. The approach of implementing total stamping out of affected herds is taken as “default” control measure in many countries, regardless of the transboundary animal disease addressed, leading to a variety of challenges when implemented. Apart from the organizational challenges and high demand for human and financial resources, the total stamping out approach puts a high burden on the livelihoods of the affected farmers. After the spread of ASF throughout the country in 2019, Vietnam changed the culling approach enabling partial culling of only affected animals in the herd, in order to save resources, and reduce the environmental impact because of the carcass disposal and allow farmers to protect valuable assets. Until now, field data comparing these disease control options in their performance during implementation has not been evaluated scientifically. Analyzing the effect of the change in a control policy, the present study concludes that partial culling can on average save over 50% of total stock with an 8-day prolongation of the implementation of control measures. With 58% of farms undergoing partial culling scoring high on a time-livelihoods matrix, while total stamping out fails to score on livelihoods, much-needed clarity on the livelihood-protecting effects of alternative culling strategies is given. In the future, this will allow veterinary authorities to adjust control measures according to differing priorities, targeting peculiarities of ASF and acknowledging resource constraints faced.

## Introduction

The expansion of a transboundary animal disease (TAD) into new territories presents an enormous challenge for veterinary services in charge of early detection and control. In addition to their routine work, they face additional tasks that need immediate attention and substantial resources. For African swine fever (ASF), as for most other TADs, the elimination of infected and contact animals forms the backbone of “default” contingency plans (1). These plans often follow the pattern: find, contain, and eliminate as fast as possible aiming to regain the disease status before the introduction.

Culling of the whole affected herds is widely considered the gold standard for ASF control in domestic pig holdings. Despite the fact that the OIE terrestrial code, as an international standard, does not require the killing of all animals on the farm under its definition of stamping out (2), the wording implies a rather stringent approach. This means that measures are usually aiming for minimal risks of infected animals escaping the culling approach. When asked recently about knowledge gaps with regards to ASF, veterinary authorities, and other stakeholders relevant to ASF control in Europe did not identify gaps in the control measures themselves (3). In line with this assumption that “we know how to handle ASF in domestic pig herds,” expert opinion on the effectiveness and practicability of control measures concluded in total stamping out of affected herds as one of the key measures to implement (4).

The aforementioned assessments follow the assumption that the complex and resource demanding implementation of total stamping out measures are adequately implemented and negative side effects are minimized successfully. Unfortunately, many countries find themselves severely challenged in this regard. This is in particular true for the aspect of timely compensation of farmers for their losses and the reduction of environmental damages when larger numbers of carcasses must be disposed of in short time. These constraints are at least acknowledged in some policy documents such as the Regional Strategy for the control of African swine fever in Africa (5). However, solutions to overcome this constraint are not highlighted. But by no means the discussion on massive stamping out as dominant tool in animal disease control is solely dominated by settings of less developed countries. The environmental impact and social acceptance, in particular with regards to sustainability in agricultural production and animal welfare, make this topic relevant around the globe (6) and alternative approaches aiming to minimize the culling are discussed (7).

The expansion of ASF into Asia in the past years (8) has had substantial impact on the livelihoods of pig producers operating in all production systems. In Vietnam, the first case was detected in a small-scale farm in the northern part of the country, in the Hung Yên province beginning of 2019 (9). Since then, the disease spread quickly throughout the country affecting farms with diverse production systems alike (8, 10, 11) and around 6 million pigs had to be destroyed because of the ASF. This severe loss of animals represented a decrease in the overall pig population of 25% by December 2019 as compared with 2018 (12). Control measures according to the national legislation were implemented in order to stop the epidemic. This included initially a total stamping out policy for affected farms that proved impossible to be implemented in many occasions because of arising challenges throughout the process. Subsequently in 7 July 2020, the Descision 972/QD-TTg of the Prime Minister in Vietnam endorsing the “National Plan for Prevention and Control of African Swine Fever for the Period 2020–2025” (13), officially changed the ASF control procedure providing the option of partial culling on infected farms. However, until now it is not documented what the concomitant effects of such a change in policy brings along for the overall disease control and the benefits of the affected farmers.

In this study, we analyze the effects of a change in the culling strategy applied in the northern Vietnam during the initial course of the ASF epidemic on the overall length of the disease event and the total loss of animals to producers. We compare the two control options applied: total culling vs. partial culling. We hereby provide evidence for adapted ASF control policies acknowledging socio-economic and environmental peculiarities of the affected countries.

## Materials and methods

Farms for this study were selected from the northern provinces of Vietnam as follows: they all had to meet the case definition of an ASF outbreak according to the Article 26 of the Law on the Animal Health (No. 79/2015/QH13 dated 19 June 2015) and were subject to disease control measures implemented by the veterinary services. Selection of farms was therefore not researcher-driven but was based on sample submission to the Department of Animal Health and Vietnam National University of Agriculture laboratories in Hanoi and Nghe An provinces.

Farm data, comprising number and type of animals, dates of disease suspicion, reporting, diagnostic and the control measures were retrieved from the official documentation of the competent authority implementing disease control measures and additional data were obtained by contacting the farmers directly.

In particular, we asked whether all the animals on an affected farm were culled or only some of them, e.g., only the sick and suspect ones (total stamping out or partial culling). There was no diagnostic test data available to evaluate whether the animals that reached slaughter from the partially depopulated farms were infected or not.

Statistical analysis and visualization of results was performed using the GraphPad Prism 9.0.0. (GraphPad Software, LLC).

For the comparison of the time span recorded for the overall control interventions between the groups of total and partial culling the non-parametric Mann–Whitney test was used after the rejection of normality by the D'Agostino–Pearson normality test.

The statistical difference between the survival rates of the animals in the two groups was calculated *via* Wilcoxon-Signed Rank test.

For the description of the correlation between the total number of animals that needed to be culled and the overall time of the control event the non-parametric Spearman correlation was used.

For the categorization of the relation between the length of the control intervention and the survival rate of animals on-farm, a Cartesian coordinate system was used where x-axis hosting the values for the duration in days is crossed by the y-axis with the survival rate at 30 days demarking twice the maximum observed time-span between infection and death of an individual pig infected with highly virulent ASF strains (14).

## Results

A total of 81 farms from 14 northern provinces (Bắc Giang; Bắc Ninh; Hà Nam; Hà Nôi; Hà Tĩnh; Hải Dương; Hải Phòng; Hưng Yên; Nghệ An; Phú Thọ; Quảng Ninh; Thái Bình; Thái Nguyên and Thanh Hóa) where included in this study. They had confirmed ASF outbreaks between early 2019 and December 2020.

In that period, the veterinary authorities recorded 51 outbreaks at community level comprising 4,088 individual pig holdings. A large number of them had very few animals. The studied farms, therefore, represent around 2% of the total farms with confirmed outbreaks occurring in this period in the target area.

**Figure 1:**
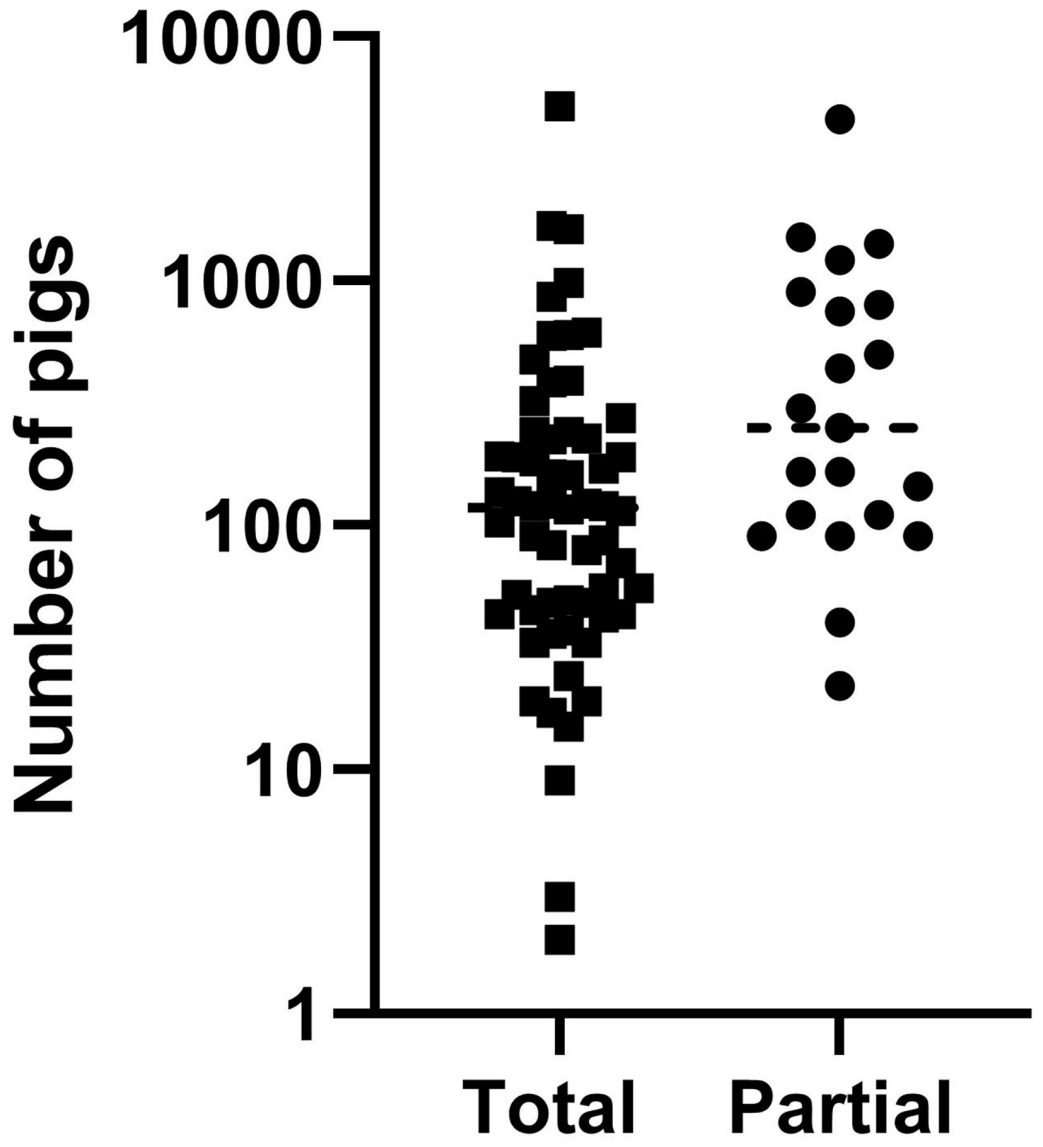
Farm size distribution separated by culling approach.

Total stamping out was performed as ASF control measure on 60 and the partial culling on 21 of them. The size of the farms included in the study ranged between 2 to 5,200 animals and 32 of the 81 farms had <100 animals representing the abundant small-scale production sector (Figure 1).

On all farms, after the disease was confirmed, control measures were implemented comprising a quarantine lifted earliest 21 days after the finalization of any culling intervention and a veterinary inspection of the premises and—if applicable—the remaining animals on farm.

**Figure 2:**
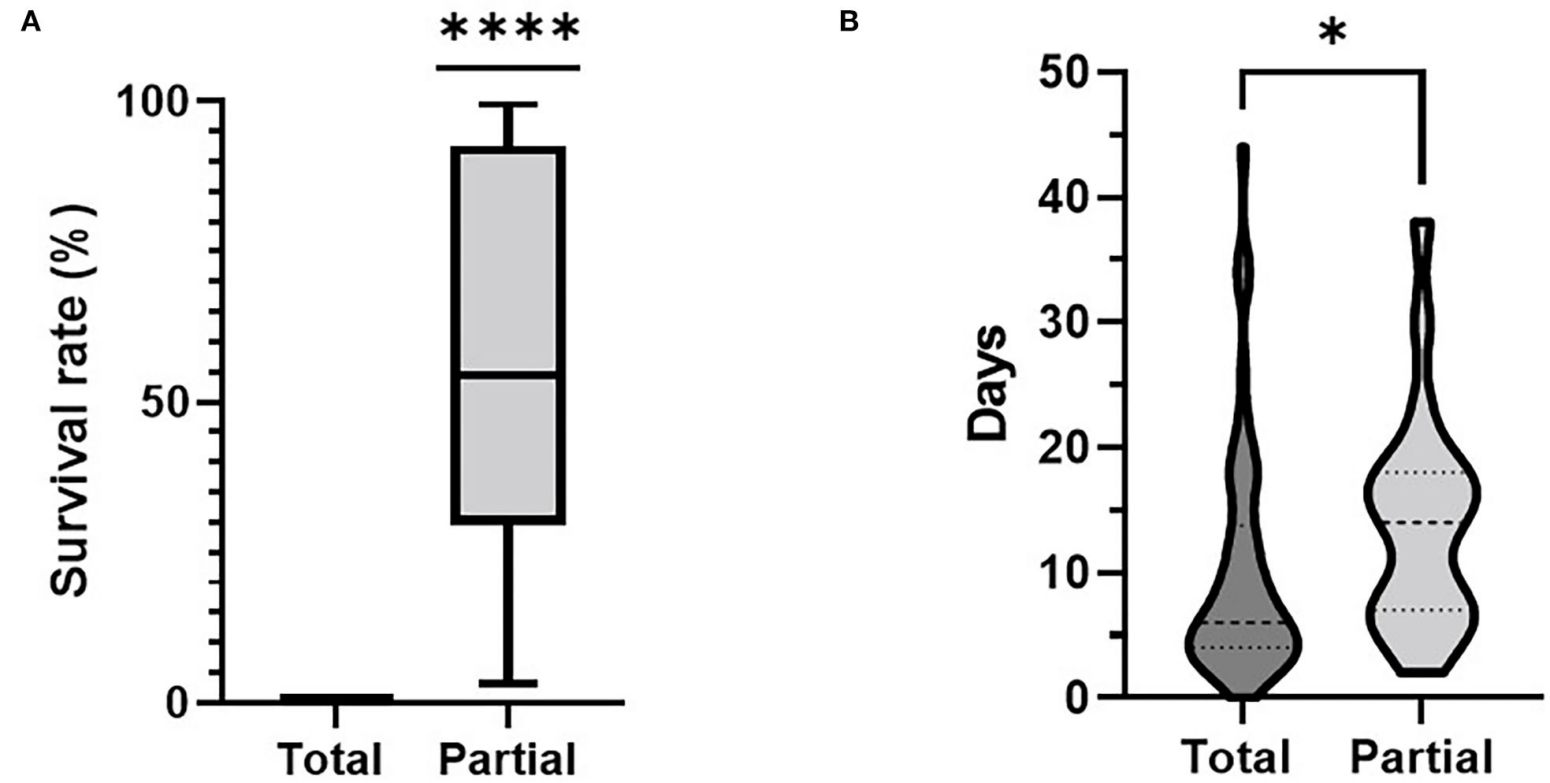
Comparison between culling approach applied and **(A)** the survival rate on farm and **(B)** time spans between onset of disease and finalization of stamping out measures. ****p < 0,0001, *p < 0,05.

The differences of the survival rate of animals observed between the two control options compared are depicted in Figure 2A. Farms with a partial culling approach had a mean survival rate of 57.57% ranging from 3 to 99%. The overall comparison of the time span needed from the documented onset of the event until the finalization of the implemented culling measures is captured in Figure 2B. Here, the time span for total stamping out ranged between 0 and 44 days with a mean value of 6 days. For partial culling, these values ranged between 2 and 38 days with a mean value of 14 days.

**Figure 3:**
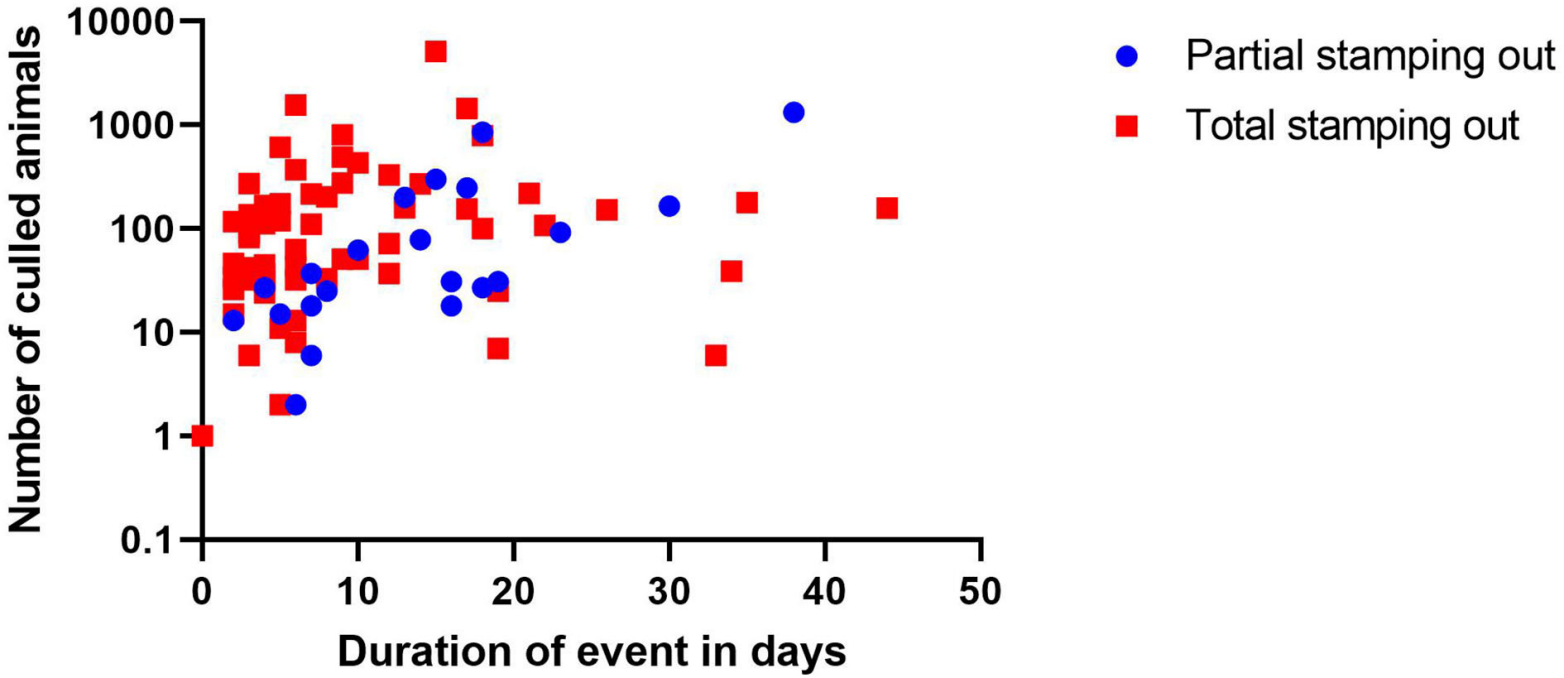
Scatterplot-depicting time span of the disease event on farm in relation to the absolute number of animals culled per farm.

Between the absolute number of animals that had to be culled per farm, depending on the culling approach, and the overall time of the control measures (Figure 3) a positive correlation is detected (*r* = 0.6880; *p* = 0.0006 for partial stamping out and *r* = 0.3225; *p* = 0.0120 for total stamping out, respectively).

**Figure 4:**
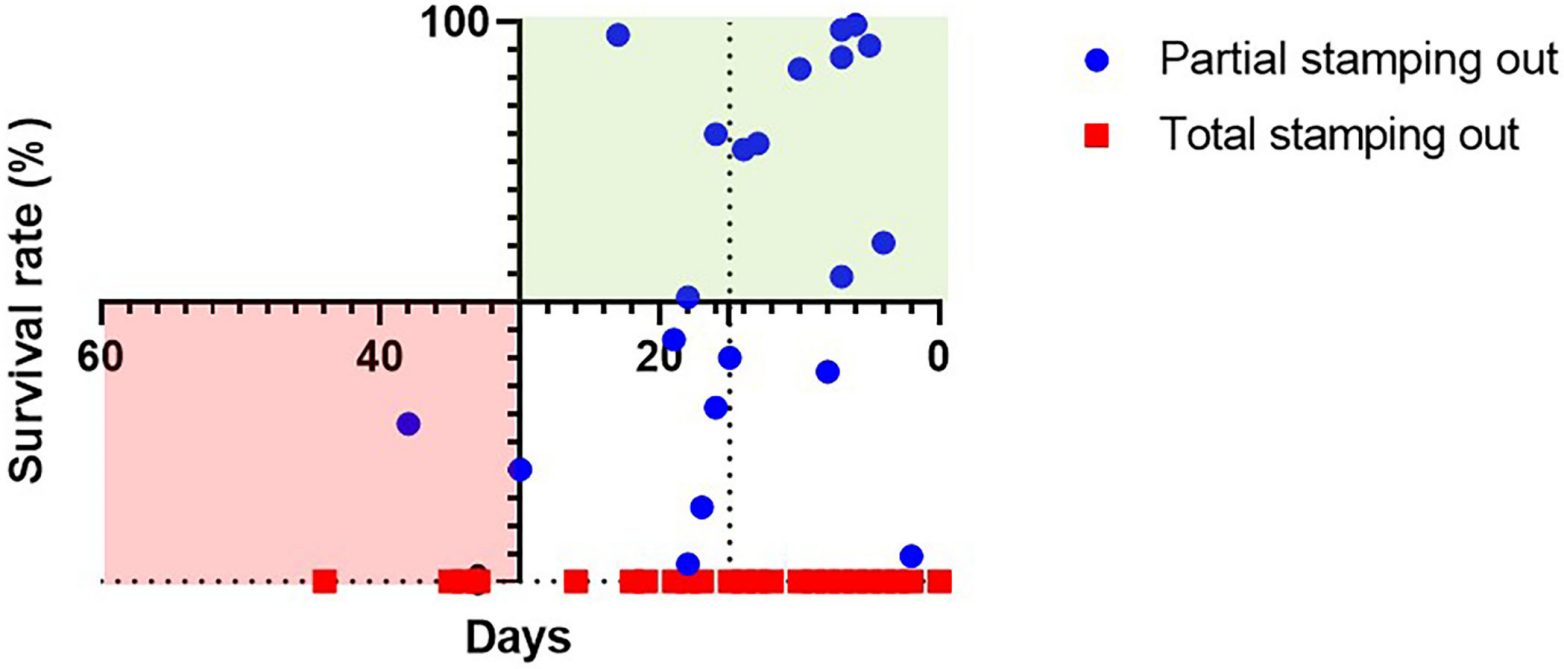
Modified Cartesian coordinate system on the relation of survival rate (in %) and the length of the disease event (in number of days) on farm. The upper right quadrant (green) standing for optimal, the lower left quadrant (red) for contrasting time efficient and livelihoods protecting interventions.

In the Cartesian coordinate system 12 out of 21 farms were partial culling was implemented are categorized in the upper right quadrant standing for optimal relation of time and livelihoods protection, none of the farms under the total stamping out fall in this category. In total, two farms of the partial culling group are categorized in the lower left quadrant standing for the least favorable relation of time and livelihoods protection. The farms with total stamping out as applied control measure remain all the categorized in the lower quadrants, 4 in the lower left and 56 in the lower right indicating the emphasis on speed over livelihoods in this approach (Figure 4).

## Discussion

The ASF epidemic in Vietnam had the highest death toll in pigs in the year 2019 when the disease spread rapidly through the country after its initial introduction. The approximately 6 million animals lost through the disease itself and the accompanying control measures meant a reduction of the total stock of around 25% and lead to a significant increase in pork prices (12). This urged for a change in control policies for the multiple reasons. In particular, the severe difficulties in the handling of a total stamping out approach triggered a change toward partial culling.

The fact that the implementation of a partial culling approach will overall reduce the number of animals culled per farm and avoids a complete destocking, forms the basis of the rationale for this alternative control measure. The data presented in this study provides much needed evidence for the possible adaptation of ASF control policies to socio-economic realities and also potential environmental constraints faced in many parts of the world without jeopardizing the effectiveness of control measures. Experiences from South Africa where selective culling has been applied show the possibility to stop ASFV circulation in the affected population in the long run (15), yet no detailed information on the timeline of the intervention or a comparative view to total culling is provided.

In our study, when partial culling was applied, in averages 58% of animals remained on farm as asset for the farmer and subsequently these animals remained in the food chain avoiding further market distraction. These results are a strong justification to be deducted for this form of control intervention that has been applied. In 2020, the documented losses in stock decreased significantly to only 1.5% of the numbers recorded in 2019 (12)—not only because of the change in control policy as the first peak of the epidemic has passed, but certainly partial culling contributed to the reduced losses. Saving assets in that dimension with alternative control options is likely to increase the acceptance of farmers toward control measures, to invest in prevention and early detection and subsequently the willingness to report disease suspicion (7). At the same time, a reduced number of culled animals will save costs for compensation for the implementing authorities and reduce the environmental burden due to carcass disposal activities. As rendering plants are not available in Vietnam and most other countries affected by the ASF pandemic, burial, burning, or composting remain as options for safe carcass disposal. All of these options have severe limitations or environmental side effects when larger numbers of carcasses need to be handled and therefore, for many veterinary authorities, the reduction of carcasses is a non-negotiable necessity regardless any effects on time-effectiveness of the disease control.

The observed range (3.6–97.8%) of surviving animals reflect the high diversity of farms, their individual farm management and the resulting disease dynamics. In best-case scenarios, the animal losses on-farm because of the ASF appear negligible, underlining the current knowledge that the disease has a lower contagiousness than often declared (16) and within farm spread *via* non-iatrogenic transmission routes is slow and can be avoided (17–19). Meanwhile, on the farms where the circumstances apparently did not allow timely interruption of infection cycles, overall losses remain high despite the selected control approach. Improved knowledge sharing and stakeholder engagement will therefore always play a crucial role in the successful involvement of farmers in disease prevention and control (20).

The duration of control interventions, to the declaration of a resolved event, certainly remains a critical dimension for disease management. However, in the present study, we can highlight that despite a statistically significant prolonged duration of the disease event with implemented partial culling, this control intervention has substantial benefits when analyzed in a time-livelihoods matrix (Figure 4). With 43% of farms undergoing partial culling that resolve the event within 15 days, it can be anticipated that no substantial within-farm infection cycles occurred during this time. For the control option of total stamping out, interventions on around 75% of farms stayed within this 15-day period underpinning the time effectiveness compared with the partial stamping out. Nevertheless, the 25% of farms exceeding this time confirm the remaining difficulties in the roll-out during outbreaks. Together it highlights that the on-farm dynamics of ASF spread—if the biosecurity management allows—can be kept minimal making continuous infection unlikely, regardless the control measure opted for. At the same time, the total stamping out option, despite being a straightforward approach, is apparently facing diverse challenges that may lead to delays in the implementation—so by no means it is the “rough but fast” solution to the problem. On the other hand, the analysis shows that a good share of affected farms addressed by a partial culling approach are among the “fast” in terms of disease control and were able to save over 50% of their pigs despite the confirmed ASF outbreak.

It remains a crucial decision for the authorities implementing ASF control measures whether the speed and rigor of implementation is the defining factor. This is considered mainly at early stages of an epidemic where a large share of the overall stock still needs to be protected. In countries such as Vietnam, where at the time of ASF introduction the setting was dominated by a high disease burden in China and the unlikely effective control in all the neighboring countries, a decision to shift in the control approach after a wide spread through the country can claim to have saved substantial resources and livelihood assets. This shift came along with the cost of uncertainty, if the prolonged and less rigor control as risk factors for disease spread, lead to outbreaks that would have been preventable through the prior control approach.

The current study did not investigate further, how extended time spans could be explained, but other factors than the mode of culling are known to influence this parameter substantially (21). As the competent authority in charge of culling is the local veterinary office in conjunction with differing other local authorities, the preparedness and also the availability of human and financial resources to implement measures will have varied throughout the study.

Not assessed are the negative side effects of total stamping out policies that are often mentioned but seldom documented (e.g., hiding of animals, emergency slaughter, and sell of pork during outbreak scenarios without veterinary inspection). As the reports of the outbreak investigations available to the study team did not obtain information on disease tracing, for none of the control approaches, we were able to analyse if they would come along with a higher risk of onward transmission and therefore a propagation of the epidemic. It has to be noted, however that solid information on secondary outbreaks and an insightful tracing of disease spread between farms is rarely achieved for any TAD epidemic. One example of documented negative consequences of culling approaches is highlighted in the immediate notification and follow-up report of Chad to OIE when facing an ASF introduction on the border with Cameroon in 2010 (22, 23). In this case, control measures have reportedly incentivized farmers to move infected animals out of control zones leading to a transboundary spread. It can at least be anticipated that any control approach implemented that reduces the loss to farmers, protects assets, and avoids driving them out of the market will increase their willingness to collaborate.

Avoiding a total depopulation of the affected holding comes along with the need for a decision on when and where to stop the culling. In ideal settings, more likely to be found in commercial farms, the combination of biosecurity, surveillance, and rapid response will allow successful implementation of a selective culling approach (7). Practical guidance on the assessment of the spread on farm based on the use of lateral-flow devices has recently highlighted that low-cost options can fit the purpose despite a reduced sensitivity over a traditional PCR-based system (24). The current study did not have access to laboratory data, neither the actual disease confirmation nor any results obtained to lift restrictions after the mandatory quarantine time of minimum 21 days after the last culling. With veterinary inspections overseeing and deciding over the control and quarantine measures, implemented within the frame of the Descision 972/QD-TTg, farms once released from restrictions were not detected again as ASF infected within the study time. In any case, passive surveillance will remain the backbone of disease control and this must be seen as additional argument to focus on control approaches protecting assets and livelihoods of affected farmers in order to encourage disease reporting.

The analyzed data is derived from the actual disease control measures, therefore having the weakness field data brings along when not following a predefined scientific approach. At the same time, it excludes the necessity to incorporate uncertainty factors for translational deductions from the obtained results, as the “real life” complexity is represented in its wide range. This mentioned complexity brings in numerous influencing factors beyond what can be scientifically evaluated but nevertheless will always comprise relevant disease drivers.

Because animals that survived in affected farms were not tested, it was not possible to assess whether the pathogen has actually been eliminated from the farm. However, the data here provide estimates of the proportion of pigs from infected farms that could still supply the market if only partial depopulation was applied. Data suggest that the impact of ASF in affected countries may be mostly associated to culling and movement restrictions, given that there is a substantial number of pigs in the affected farms that would be able to survive and consume locally without further spread of the disease. Although partial culling may represent challenges for the control of the disease at a country level, given that pork from persistently infected animals may still enter the value chain, social, animal welfare, food security, and economic factors may prompt the consideration of partial culling as a strategy to mitigate the impact of disease, even at the cost of, potentially, creating conditions for disease endemicity.

From the obtained results we can conclude that partial stamping out represents a valuable alternative for ASF control with several benefits to farmers and veterinary services, in particular, if resource constraints are heavily impacting the response capabilities. However, it does not release the involved parties from the need to apply strict quarantine, animal movement control, and biosecurity protocols during outbreak scenarios regardless of the control option applied. This is of upmost importance for all occasions where prolonged control measures are observed, as here we encounter the highest risk of disease spread. In addition, the evolving biology of circulating ASFV strains must be considered in the equation as strains with the lower virulence and potential carrier animals among disease survivors (25) are likely to complicate the partial culling approach when implemented without supporting diagnostic tools. The benefits to the individual farmer of partial culling as chosen ASF control measure are evident and highlight the potential pro-poor character of this approach. In particular, countries with limited resources for compensation and larger fraction of farmers with lower incomes, are encouraged to revisit their control plans accordingly and to allow situation-tailored interventions.

## Data availability statement

The original contributions presented in the study are included in the article/Supplementary material, further inquiries can be directed to the corresponding author.

## Author contributions

BN coordinated and conducted field data acquisition and contributed to data analysis and to the drafting of the manuscript. PP advised on field data acquisition and analysis and provided scientific guidance during the drafting of the manuscript. VC, NA, and DD assisted in field data acquisition and provided scientific guidance during the drafting of the manuscript. KDe advised on data analysis and provided scientific guidance. KDi established the concept of the project, contributed to data analysis and drafted the manuscript. All authors contributed to the article and approved the submitted version.

## Conflict of interest

The authors declare that the research was conducted in the absence of any commercial or financial relationships that could be construed as a potential conflict of interest.

## Publisher's note

All claims expressed in this article are solely those of the authors and do not necessarily represent those of their affiliated organizations, or those of the publisher, the editors and the reviewers. Any product that may be evaluated in this article, or claim that may be made by its manufacturer, is not guaranteed or endorsed by the publisher.
